# Zirconium-Based Metal Organic Frameworks for the Capture of Carbon Dioxide and Ethanol Vapour. A Comparative Study

**DOI:** 10.3390/molecules26247620

**Published:** 2021-12-15

**Authors:** Meryem Saidi, Phuoc Hoang Ho, Pankaj Yadav, Fabrice Salles, Clarence Charnay, Luc Girard, Leila Boukli-Hacene, Philippe Trens

**Affiliations:** 1 Institut Charles Gerhardt des Matériaux (ICGM), Univ. Montpellier, CNRS, ENSCM, 34090 Montpellier, France; meyriem.saidi@gmail.com (M.S.); hoangphuocho@gmail.com (P.H.H.); pankaj.yadav@umontpellier.fr (P.Y.); fabrice.salles@umontpellier.fr (F.S.); clarence.charnay@umontpellier.fr (C.C.); 2 Department of Chemistry, Tlemcen University, Tlemcen BP 119, Algeria; leila.bouklihacene@univ-tlemcen.dz; 3 Institut de Chimie Séparative de Marcoule (ICSM), Univ. Montpellier, CNRS, ENSCM, CEA, 30207 Bagnols sur Cèze, France; luc.girard@enscm.fr

**Keywords:** adsorption, metal organic framework, carbon dioxide, ethanol

## Abstract

This paper reports on the comparison of three zirconium-based metal organic frameworks (MOFs) for the capture of carbon dioxide and ethanol vapour at ambient conditions. In terms of efficiency, two parameters were evaluated by experimental and modeling means, namely the nature of the ligands and the size of the cavities. We demonstrated that amongst three Zr-based MOFs, MIP-202 has the highest affinity for CO_2_ (−50 kJ·mol^−1^ at low coverage against around −20 kJ·mol^−1^ for MOF-801 and Muc Zr MOF), which could be related to the presence of amino functions borne by its aspartic acid ligands as well as the presence of extra-framework anions. On the other side, regardless of the ligand size, these three materials were able to adsorb similar amounts of carbon dioxide at 1 atm (between 2 and 2.5 µmol·m^−2^ at 298 K). These experimental findings were consistent with modeling studies, despite chemisorption effects, which could not be taken into consideration by classical Monte Carlo simulations. Ethanol adsorption confirmed these results, higher enthalpies being found at low coverage for the three materials because of stronger van der Waals interactions. Two distinct sorption processes were proposed in the case of MIP-202 to explain the shape of the enthalpic profiles.

## 1. Introduction

In recent decades, the consumption of fossil fuels has increased alarmingly to meet world population needs, in terms of economy and industry. The latter emits a large amount of CO_2_ into the atmosphere, which causes major climatic issues and therefore a real danger for our environment. Indeed, according to the literature, the concentration of CO_2_ in the atmosphere has increased considerably, passing from 340 ppm in 1980 to 408 ppm in 2019 [1]. However, other threats also affect our environment, namely volatile organic compounds (VOCs) [2]. A majority of these products have been designated as factual air pollutants [3,4,5], knowing that the Health Effects Institute has plainly designated air pollution as the fifth risk factor for mortality in the world in 2019 [6].

To solve these environmental issues, it is important to find appropriate techniques for capturing CO_2_ and volatile organic compounds that can be beneficial in agriculture and industry [7]. The adsorption technology remains by far the best method to remove and capture VOCs and CO_2_, for its ease and flexibility [1,8]. However, it is necessary to choose the appropriate sorbent which represents the most important factor of this technological approach, as its efficiency has a direct impact on investment and operating costs [2].

Activated carbon (ACs) and zeolites are very common adsorbents which have therefore been extensively studied for this purpose. Activated carbons have a large specific surface area and a well-developed porous structure, which justify the interest in the various works carried out. However, many drawbacks can be mentioned concerning this porous material. For instance, it has been proven that ACs suffer from a regeneration problem, which presents a significant weakness on a large scale; as well as a flammability risk, particularly through exothermic adsorption [9,10,11,12]. This is why, because of its very low cost, it is usually replaced after use. Additionally, Lai et al. noted that its amorphous structure limits the improvement of its performance for VOC removal [13]. Concerning carbon dioxide capture, Ghanbari et al. mentioned the low sorption capacity of ACs at ambient temperatures due to the lack of specific ACs/CO_2_ interaction as the surface functions of ACs are mostly acidic functions [1].

Other very common sorbents are the zeolites family, which possess a high specific surface area, well-defined pore structure, and great thermal stability. Some studies highlighted some disadvantages presented by zeolites, including a rather complex synthesis and high-cost reactants such as tetraethyl orthosilicate and cetyltrimethyl ammonium bromide that must be removed after synthesis, usually by total oxidation. It must be underlined that some zeolites can be found readily in the nature such as chabazite or mordenite. However, their structures are therefore defined, which raises a question about their versatility, especially when specific interaction are required. Additionally, some of them proved to be unstable in humid atmospheres, which is a clear limitation for real applications where water is always present [14,15].

As alternatives, metal–organic frameworks (MOFs) are a new class of hybrid porous adsorbents constructed from inorganic subunits, linked by organic ligands (carboxylates, imidazolates, or phosphonates). They have several key characteristics such as large specific surface areas, a wide variety of structures, and large pore volumes [16]. Many studies have pointed out that some MOFs also exhibit excellent VOCs and CO_2_ adsorption capacities [1,2,17,18,19,20,21,22,23]. Furthermore, unlike MOF-5 or HKUST-1 [24,25], some MOFs present high stability in water, which is a prerequisite for VOCs or CO_2_ capture in ambient conditions [22,26]. It is the case of MOFs based on ‘hard’ metal ions such as Zr^4+^, Ti^4+^, Fe^3+^, Cr^3+^ bound to organic carboxylate ligands [27]. As the ligands can be functionalized before the MOF syntheses or using post-functionalization approaches, it is therefore possible to design specific materials that are able to interact with particular sorbates [26]. Vellingiri et al. studied the toluene adsorption capacity of four MOFs (MOF-5, MIL-101 (Fe), MOF-199, and ZIF-67). Beyond the contrasted sorption capacities of these MOFs [28], these authors pointed out that the carboxylic group in MOF-199 and the presence of nitrogen atoms in ZIF-67 contributed considerably to the adsorption capacity, via hydrogen bonding. Another example has been recently published by He et al. These authors emphasized the importance of nitrogen function in CO_2_ sorption processes [18]. They showed that an acid-base bond was formed by the reaction between CO_2_ and the basic nitrogen sites of MOFs. This conclusion is consistent with the recent work of Ma et al. [29]. These authors synthesized nanoporous carbons (MUCT) from MOF-5 and urea, leading to a CO_2_ adsorption capacity of 3.71 mmol g^−1^ at 273 K under atmospheric pressure. They also concluded that the adsorption efficiency was tightly related to the presence of nitrogen atoms and alcohol functions in the MUCT structure.

Amongst MOFs exhibiting great textural and chemical stability, UiO materials centered around Zr are therefore promising stable materials for sorption purposes in industrial conditions. In this framework, Vellingiri et al. compared the sorption capabilities of UiO-66-(Zr) and UiO-66-(Zr)-NH_2_ in the case of VOC capture [26,30,31,32]. These authors emphasized the importance of the amino group in the UiO-66-(Zr)-NH_2_ cavities, which led to a clear improvement in the sorption of toluene compared to the original UiO-66-(Zr). If the functional group in the cavities of the MOFs are of prime importance in the frame of sorption, Ramsahye et al. demonstrated that confinement effects could also be responsible for an enhancement of the sorption, both in terms of the adsorbed amount at saturation of the porosity and in terms of the sorbate/MOF interaction [33]. Three MOFs were compared, including UiO-66-(Zr), and clear differences could be established in terms of sorption selectivity and capacity, thus exemplifying the influence of the pore topology and therefore confinement effects. In MOFs, structural defects are also known to confer specific interaction useful for catalysis and separation purposes [34]. These defects can be provided in the structures mainly through missing linkers between building units. However, their number, their location, and their distribution are often difficult to define [35]. Defects induce several beneficial consequences by (i) increasing the pore size, (ii) creating space around the metal centers available for possible adsorbates, and (iii) giving rise to additional sites where sorption can also occur. In terms of their nature, these missing ligands induce coordinatively unsaturated sites in the material, resulting in open Lewis acid sites which can therefore play a major role in sorption purposes [36]. Many articles have reported on CO_2_ and VOCs adsorption by UiO-66-(Zr), but very few articles tackled CO_2_ and VOCs adsorption properties of other Zr-MOFs, especially with a smaller linker length and therefore smaller pore size. Very recently, the sorption properties of the three Zr-MOFs have been evaluated in different studies, principally for CO_2_/N_2_ or CO_2_/CH_4_ sorption selectivity. Chen et al. explored the ideal permeability-selectivity of the novel MOF-801/PIM-1 mixed-matrix membranes for enhanced CO_2_/N_2_ separation performance. It was concluded that the mixed membrane has high permeability and selectivity to carbon dioxide [37]. Sun et al. explored a new mixed-matrix material (MOF-801 incorporated in a polyether block amide (PEBA) mixed-matrix composite membrane) for CO_2_ capture. The new mixed material showed a remarkable improvement both in CO_2_ permeance and CO_2_/N_2_ selectivity compared with the pristine PEBA membrane [38]. Iacomi et al. presented a theoretical and experimental investigation on the propane and propylene adsorption in MOF-801, showing the preference for propane over propylene, thus suggesting the potential applicability of the Zr-fum-MOF in a propane/propylene separation [39]. Lv et al. studied the separation performance of MIP-202 for CO_2_/CH_4_ and CO_2_/N_2_ mixtures. MIP-202 exhibited an ultrahigh CO_2_/CH_4_ and CO_2_/N_2_ adsorption selectivities. The interpretation given was the fact that CO_2_ molecules could diffuse in the pore walls of the large cages which have a higher polarity while CH_4_ or N_2_ molecules could diffuse in the pore walls of the small cages which have a lower polarity [40]. The improved synthesis of Muc-Zr-MOF and its textural properties was investigated by Buragohain et al. [41]. This study highlighted the potential of this material for sorption of larger sorbates.

**Figure 1:**
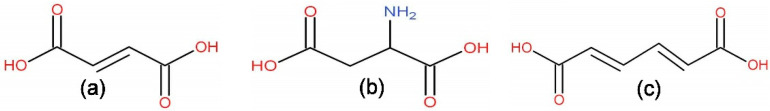
(**a**)-fumaric acid; (**b**)-aspartic acid; (**c**)-muconic acid.

In the frame of our study, we therefore decided to focus on these three zirconium-based MOFs constructed using fumaric acid, aspartic acid or muconic acid as linkers (see Figure 1) for the adsorption of ethanol vapour and CO_2_.

**Figure 2:**
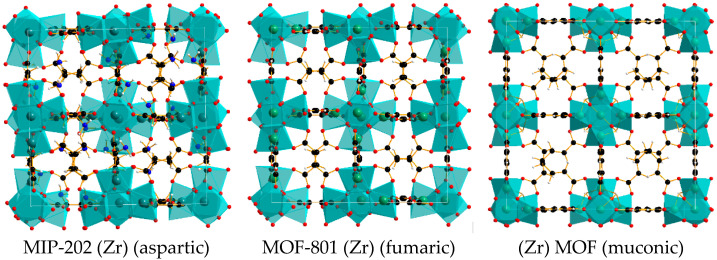
Structures of the three MOFs investigated (black: carbon, red: oxygen, green: Zr, blue: nitrogen, white: hydrogen).

MOF-801 is formed from Zr^4+^ ions and fumaric acid ligands, forming octahedral clusters positioned at the top of a cubic lattice (Figure 2) [42,43]. This MOF is already known for its high water stability [44] and its potential as a water harvesting device [45]. MIP-202 is formed from Zr^4+^ ions and L-aspartic acid ligands. It is isostructural to MOF-801. It is obtained by green synthesis (use of water as a solvent) and has several advantages, such as: it is a rather affordable linker, has a straightforward synthesis route without the use of any modulator [40,46], and has a good synthetic yield. More importantly, the ligands of this MOF bear NH_2_ functional groups, which have been shown to enhance the adsorption efficiency of acidic species, *vide supra*. More precisely, in acidic conditions for the synthesis, the linker groups bear -NH_3_^+^ electrically equilibrated by Cl^−^ [44]. The last MOF evaluated is Muc-Zr MOF (see Figure 2). This MOF formed from Zr^4+^ ions and trans muconic acid ligands is isostructural to UiO-66-(Zr) [41]. This MOF possessing olefinic linkers therefore has larger micropores compared to the other microporous materials studied in this work. In the following, we performed the synthesis of the three Zr-based MOFs, and after thorough characterization, we measured their gas adsorption (CO_2_ and ethanol) capacities in order to explore the influence of the MOFs’ morphology and the nature of the ligands over their gas sorption behavior.

## 2. Results and Discussion

### 2.1. Characterization of MOFs

**Figure 3:**
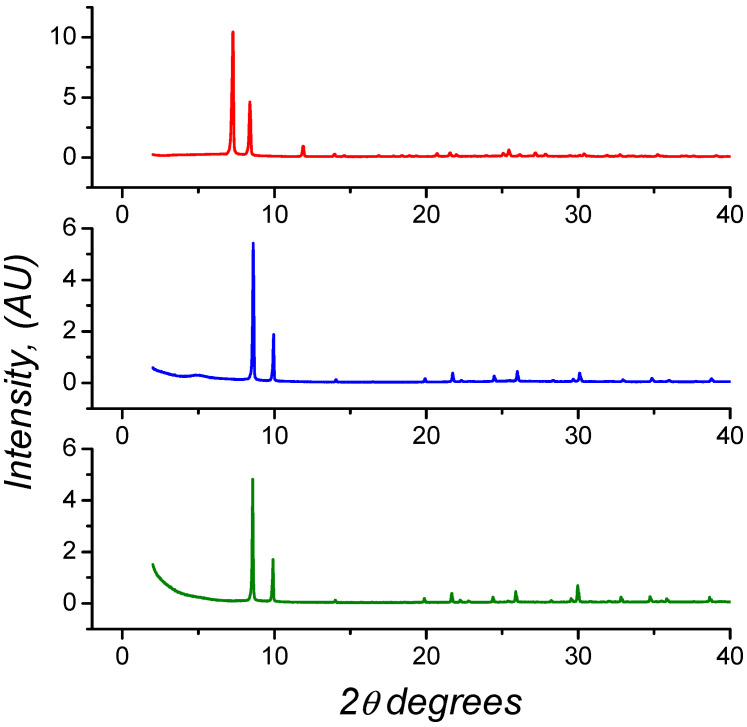
X-ray diffraction patterns of the synthesized MOFs: (**top**) Muc-Zr MOF; (**middle**) MIP-202 MOF and (**bottom**) MOF-801.

The XRD patterns of the synthesized solids shown in Figure 3 are very similar to those documented in the literature [40,42]. MOF-801 and MIP 202 are isostructural, with the main peaks situated at 2θ equal to 8.77, 10.09, 30.10 and 8.65, 10.07, 30.10, respectively.

These patterns can be indexed in the cubic space group Pn3. In this space group, the crystal structure is characterized by an octahedral cavity and two tetrahedral cavities. The latter are crystallographically independent and differ in size (5.6 Å and 4.8 Å diameter). The diameter of the octahedral cavity is 7.4 Å [40,41,42,43]. These values are in good agreement with the pore size distribution obtained from the theoretical structures optimized by DFT (see below). It should be noted that the precision of the calculations leads to the observation of two separated peaks (above 3 Å for Muc-Zr and lower than 3 Å for MIP-202 and MOF-801) in some range which correspond to similar pores. MIP-202 and MOF-801 have almost identical crystallographic parameters (*a* = 17.8348 Å for MOF-801 and *a* = 17.8260 Å for MIP-202), which is consistent with the length similarity of the two linkers. However, the environment of the cavities of MIP-202 is very different to that of Zr-fumarate as all amino groups are located in the inner walls of the pores of MIP-202 [44]. According to Lv et al., the two types of cages (large octahedral and small tetrahedral cages) are in a 5:8 large cages to small cages ratio [40]. The X-ray powder diffraction (XPRD) of Muc-Zr MOF shows main diffraction peaks located at 7.27, 8.39, and 11.09 2θ values. These values are almost identical to those found for UiO-66 (Zr) [47]. Muc-Zr MOF is therefore isostructural to UiO-66 (Zr) with a cubic structure made of octahedral and tetrahedral microporous cages (7.5 Å and ~12 Å). From the theoretical pore size distribution, the two pores give similar pore sizes due to the spherical model used to probe the cavity size, and therefore very different from octahedral or tetrahedral shapes (see below).

**Figure 4:**
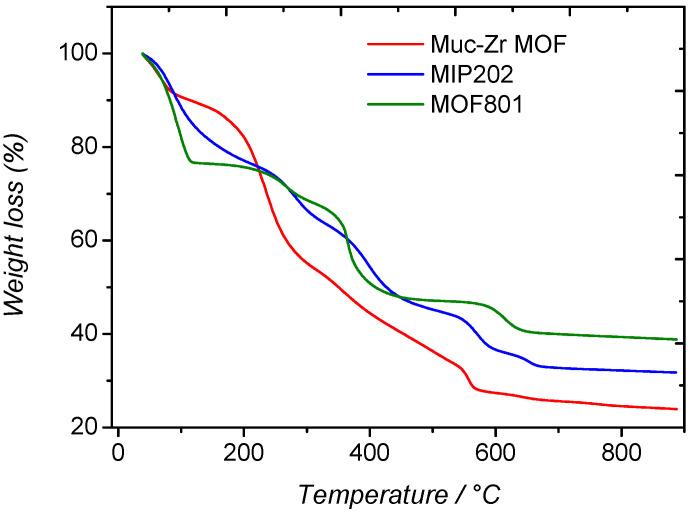
Thermograms of MOFs: (green) MOF-801, (blue) MIP-202, and (red) Muc-Zr MOF.

The thermogravimetric analyses of the prepared MOFs are compared in Figure 4.

We note that the three MOFs were prepared using different ligands and modulators, thus inducing differences in terms of weight losses and thermal stability. However, general trends can be observed. At low temperatures, adsorbed water is removed, followed by the decomposition of the ligands and the formation of zirconium oxide at high temperatures. Concerning the water removal, its extent differs between Muc-Zr MOF on one side, and MIP-202 and MOF-801 on the other side. In the latter cases, the weight loss is around 23% up to 150 °C, whereas in the case of Muc-Zr MOF, the water removal is only ~9% at 120 °C. It can be deduced that Muc-Zr MOF is less hydrophilic than the other MOFs. These weight loss differences could originate from the drying stage of the materials; however, they have been placed in the same climatic oven for the same duration. These weight losses are consistent with former studies, which supports our suggestion [28,40,48,49].

In this temperature region, the weight loss is much faster in the case of MOF-801, compared to the other materials. This is likely due to the presence of physisorbed water in the mesopores of this material (see below). As confinement effects are directly related to the size of the pores, it can be deduced that water desorption can take place faster in mesopores compared to that observed for the microporous muc-Zr MOF and MIP-202 [48,49].

At higher temperatures, the decomposition of the ligands takes place between 250 and 450 °C, which agrees well with the known decomposition of bulk ligands that occurs at 290–400 °C. At around 550–700 °C, a step can also be assigned to the decomposition of the ligand evolving from the material as CO_2_ formed from carbonate ions bonding to zirconium cations [50]. However, in the case of MOF-801 and MIP-202, an additional sub-step is present at around 250 °C. This sub-step is not present in the thermal analyses already published, which suggests that some impurity could be present in our materials. Even if the materials were carefully washed and dried before characterization procedures, some DMF or formic acid molecules could have remained trapped in the micropores of Muc-Zr MOF and MOF-801. As formic acid and DMF have a bulk vaporization temperature of 101 °C and 153 °C, respectively, the weight losses observed at 250 °C could be related to the removal of these species, which are strongly bound to the surface of the micropores of the MOFs.

The purity of the materials was further investigated using Fourier transform infrared spectroscopy. The transmittance spectra are shown in Figure S2. They are consistent with published spectra [41,44,48]; however, there are some extra bands that require some attention. In the case of MOF-801, whereas the band located at 1399 cm^−1^ corresponds to the C-C bond of the fumaric acid, the bands situated at 1600 cm^−1^ and 1255 cm^−1^–1102 cm^−1^ correspond to the C=O bond likely assigned to the DMF solvent and to the C=O bond attributed to the formic acid used as modulator, respectively [50]. The presence of DMF in Muc-Zr MOF can be found at 1650 cm^−1^ along with the bands related to muconic acid in the region 1615 cm^−1^–1385 cm^−1^ (C-O bonds of trans, trans muconic acid), and at lower wavenumbers for the C=C bonds (810, 740 and 710 cm^−1^).

MIP-202 was prepared in aqueous solution without modulator. The corresponding infrared spectrum mainly shows the presence of aspartic ligands, the transmittance band at 1652 cm^−1^ corresponding to its C=O bond, the asymmetric bands between 3490 and 3380 cm^−1^ corresponding to the amino function vibration, whereas the bands located in the region 1220 cm^−1^–1020 cm^−1^ can be assigned to the C-N stretching. From these observations, it can be concluded that formic acid and DMF can be found in these materials as traces.

**Figure 5:**
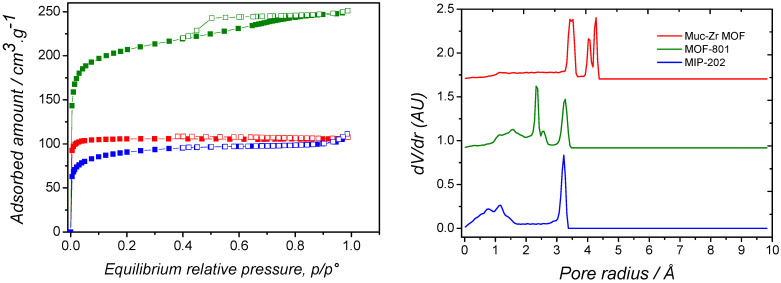
(**Left**) Nitrogen sorption isotherms. (Green) MOF-801, (red) Muc-Zr MOF, (blue) MIP-202. Open symbols correspond to the desorption branches of the sorption isotherms. (**Right**) Pore size distribution of (red) Muc-Zr MOF, (green) MOF-801, (blue) MIP-202.

The textural properties of the prepared materials were evaluated by nitrogen adsorption at 77 K. The corresponding sorption isotherms are shown in Figure 5 (left) and the textural parameters are gathered in Table 1. These were calculated using the BET method. The average pore diameter of the different materials was derived using the BJH method on the desorption branch of the sorption isotherm, taking *p/p*° = 0.85 as the starting point for the derivation. The external surface areas were calculated using the *t*-plot method.

They are type I sorption isotherms, according to the IUPAC classification, typical for microporous materials. After a strong nitrogen uptake at very low relative pressure, corresponding to the filling of the micropores, the sorption isotherms exhibit rather flat plateaus. This is the indication that the external surface of the materials is moderate compared to the total surface. This is especially the case of Muc-Zr MOF whose external surface area is only 14 m^2^·g^−1^ (see Table 1). The adsorbed amount at saturation observed in the case of MOF-801 is very high, compared to the other microporous materials. Additionally, a slight inflexion of the adsorption isotherm curve can be observed at *p/p*° = ~0.7. This can be related to the occurrence of some large mesopores. These mesopores could have been produced by the presence of defects, or lacunaries, in MOF-801. As the structures of MOF-801 and MIP-202 are isoreticular, the difference in specific surface areas (920 m^2^·g^−1^ and 405 m^2^·g^−1^, respectively) could originate from the different particle size leading to different external surface areas (205 m^2^·g^−1^ and 106 m^2^·g^−1^, respectively) but also from these mesopores. In addition, the presence of Cl^−^ in the structure of MIP-202 can prevent from the entrance of N_2_ molecules and therefore limit the accessible specific surface area. The high specific surface area of MOF-801 is confirmed by Ke et al. [51]. However, in that study, the specific surface area was not discussed in terms of mesoporosity. The presence of mesopores in our material is confirmed by the wide H2 hysteresis loop only observed for this material, and their average size is 68 Å.

**Figure 6:**
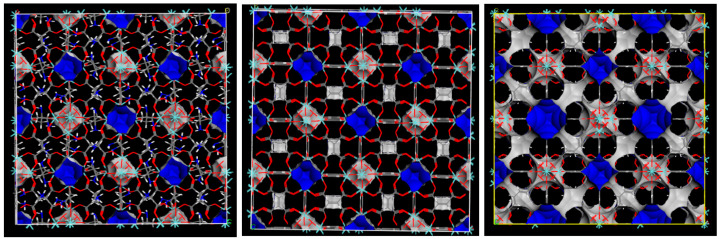
(**Left**) MIP-202, (**middle**) MOF-801 and (**right**) Muc-Zr MOF structures with the specific surface area accessible to N2 (blue and grey).

The micropores size distribution could also be determined by molecular simulations. The results are presented in Figure 5 (right). Muc-Zr MOF shows two distinct types of micropores, which have radii of 4.27 Å and 3.52 Å. In contrast, the isostructural MOF-801 and MIP-202 have a similar main pore size, located at a radius of 3.27 Å. These results can be related to the larger size of the muconic ligand compared to the fumaric or the aspartic ligands. At lower pore radius, wide and rather flat peaks could be related to the windows of the cavities, which would therefore range between 2 Å and 3 Å diameter. It can be also noted that these calculations were performed by assuming perfect crystals, and therefore pure microporous materials. It is therefore not surprising at all to see no mesoporosity in the case of MOF-801. However, the existence of 2.3–2.4 Å radius pores (corresponding to the pores in the Zr cluster) should be excluded from the discussion in MOF-801 and MIP-202. In order to compare the three structures, the accessible specific surface area can be visualized in the snapshots of the Figure 6. It can be seen that the nitrogen molecule can visit the different pores of Muc-Zr, while it is more localized in MIP-202 and MOF-801. Using the strategy proposed by Düren et al., it is possible to extract from the crystal structures the specific surface area and the pore volume for each solid. The corresponding data are reported in Table 2.

The comparison between experimental and theoretical values unambiguously confirms that the Muc-Zr MOF should contain some impurities since the theoretical values are higher than the experimental ones. This could be due to some residual DMF, used as a solvent, as already shown by Guillerm et al. [47]. In their study, they found a specific surface area of 705 m^2^·g^−1^ after activation at 250 °C. At this temperature, however, the authors concluded that the material already underwent some thermal degradation, leading to lower specific surface areas. In contrast, for MOF-801 and MIP-202, the values are in the same order of magnitude and the differences can be explained by the flexibility of the framework (rotations of the ligands, relaxation of the chemical bonds, etc.) allowing to accommodate N_2_ molecules in the experiments while the simulations fail to reproduce such behavior. It follows that the order of pore volume is the following: MIP-202 < MOF-801 < Muc-Zr. Additionally, the differences in pore volume between experimental and theoretical values in MIP-202 can be explained by the presence of the extra-framework anions (Cl^−^) which can prevent the entrance of N_2_ in some pores.

The morphology of the prepared materials was investigated using SEM. The corresponding pictures are presented in Figure S3. MOF-801 and MIP-202 exhibit smaller particle size compared to Muc-Zr MOF, which is consistent with our conclusions from experimental nitrogen adsorption. Additionally, the latter has aggregated particles which lowers its accessible external surface even more. Even if some large square-based bipyramids can be seen, most of the crystalline particles likely underwent some coalescence during their synthesis. By comparing the different solids, similar particles sizes can be noticed in the case of MIP-202 and MOF-801. However, in the latter case, isolated particles appear more distinctive, suggesting less aggregation and, therefore, a higher external surface area.

### 2.2. Carbon Dioxide Adsorption

Some recent sorption studies investigated the possibility to adsorb CO_2_ or VOCs at rather low pressure and room temperature [17,21]. The reason behind this is probably to focus on realistic conditions as in the atmosphere these pollutants are diluted in air at room pressure. The three investigated MOFs were therefore tested in these greener conditions, and their performances were evaluated.

**Figure 7:**
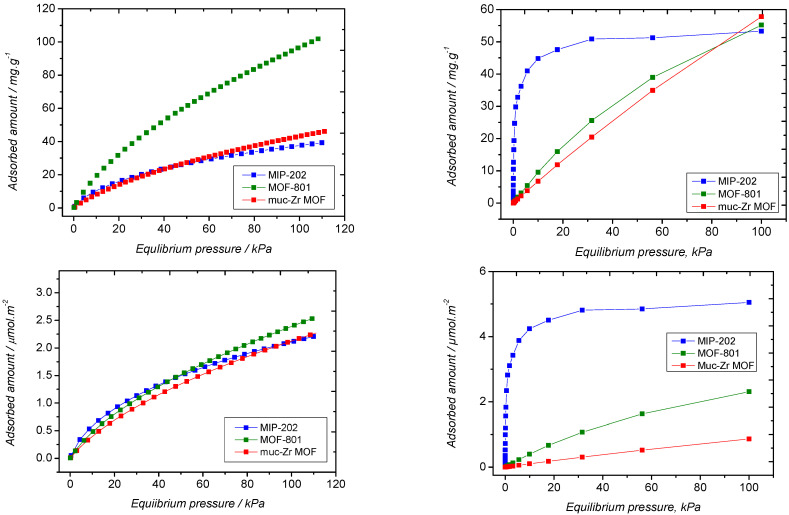
Carbon dioxide adsorption isotherms at 298 K by the prepared MOFs. (Red) Muc-Zr MOF, (green) MOF-801, (blue) MIP-202; (**top left**) experimental data, (**top right**) predicted data. Experimental data reported as per square meter. (**bottom left**) Simulated data reported as per square meter (**bottom right**).

These experiments were performed using unusually large amounts of adsorbents (~100 mg), as moderate adsorption was expected at ambient pressure due to the rather moderate interaction involving CO_2_ with MOFs [29,52]. Additionally, when the interactions ruling the sorption process are close to kT, room temperature is a parameter that classically penalizes sorption. The carbon dioxide adsorption isotherms are presented in Figure 7. The shape of the sorption isotherms follows the same trends, regardless of the material studied. As expected, the CO_2_/MOF affinity is not very high, as denoted by the rather moderate slope at low relative pressure. Additionally, the inflexion of the curves, which also reflects this affinity, is not very marked. This is more especially true in the case of Muc-Zr MOF and MOF-801, which suggests that these materials weakly interact with carbon dioxide molecules. It can be noted that up to 20 kPa in the case of MOF-801 and Muc-Zr, the adsorbed amount increases in linear fashion versus the relative pressure. This is an indication that the surface appears as homogeneous for CO_2_ molecules. In contrast, in the case of MIP-202, there is a noticeable inflexion in the sorption isotherm located at *p* = 10 kPa. This inflexion can be interpreted as the change between two sorption regimes [53]. This shows the influence of high interaction sites (extra-framework anions (Cl^−^) and amine functions) as well as the impact of the framework on the CO_2_ adsorption mechanism.

It can be deduced that CO_2_ has two different sorption regimes in MIP-202. In MIP-202, extra-framework anions and amino groups borne by the aspartic acid ligand likely are the main interaction for carbon dioxide. Once these sites are saturated, sorption can occur at higher pressures on the other sites in this MOF. In terms of theoretical adsorbed amounts, MOF-801 exhibits a slightly better sorption capacity, compared to MIP-202, which is consistent with the similar theoretical pore volumes (0.32 cm^3^/g for MOF-801 and 0.27 cm^3^/g for MIP-202. This is also consistent with the difference observed in terms of specific surface areas of these two materials. The morphological factors invoked above are therefore also responsible for the higher sorption capacity, (i) lower particle size, and (ii) higher external surface area. In general terms, the adsorbed amounts obtained are consistent with the literature. For instance, Grajciar et al. obtained a theoretical CO_2_ adsorbed amount of ~130 mg.g^−1^ in CuBTC at 1 bar. This result is not far from our own results as CuBTC has a higher specific surface area [54]. Sun et al. prepared a PEBA matrix in which MOF-801 nanocrystals were incorporated. They reported an adsorbed amount of 5 cm^3^·g^−1^ at *p/p*° = 0.1 when CO_2_ was adsorbed on the MOF-801 crystals alone, which is less than the adsorbed amount obtained in our work [38]. Our results are also consistent with the theoretical sorption isotherms (Figure 7, top right). Indeed, the comparison of saturation capacities for Muc-Zr-MOF and MIP-202 between experimental and theoretical results show a very good agreement, while those for MOF-801 are drastically different due to the presence of mesopores. Differences between experimental and theoretical results at low loadings for MIP-202 regarding the highest affinity for CO_2_ can be explained by the use of extra-framework anions (Cl^−^) which are free of solvent and therefore directly available for adsorption in molecular simulations, while they can be already impacted by impurities (water or solvent molecules) present in the pores, which is consistent with its low experimental pore volume. However, the fair consistency between experiments and simulations validates the force fields developed here for the solids. The interaction involved can be better appreciated by plotting the adsorbed amount per square meter in order to discard the influence of the extent of the surface area onto the sorption properties of the materials (Figure 7, bottom). The adsorption isotherm obtained with MIP-202 exhibits a higher affinity, and the inflexion corresponding to the change of sorption process is more pronounced than in the case of the other materials. It is even more striking when looking at Figure 7 (bottom right), where the simulated sorption isotherms have plotted. The differences observed between the adsorbed amounts at 100 kPa can be related to the strong affinity of MIP-202 in contrast to other MOFs (as illustrated below by the theoretical adsorption enthalpy at low coverage), the effect of the confinement (MOF-801 has smaller pores compared to MUc-Zr-MOF) and probably the density/accessibility of the more energetical sites (strongly dependent from the size of the linkers). This representation therefore highlights the interpretations suggested above. The sorption capabilities of our Zr-based MOFs compared well with those of other MOFs, as highlighted by Li et al. and Ghanbari et al. in their reviews [1,17].

**Figure 8:**
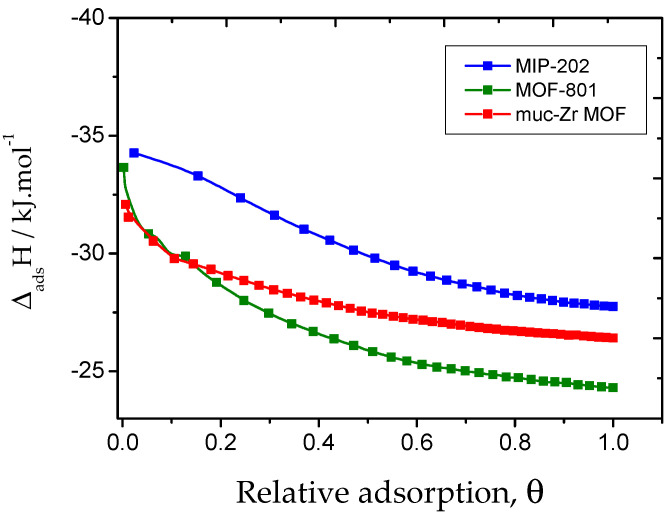
Isosteric heat of adsorption of CO_2_ by the three MOFs obtained at temperatures of 273 K and 298 K.

In terms of interactions, we also derived the isosteric heat of adsorption for the different systems, as shown in Figure 8.

Experimentally, the isosteric heats of adsorption were derived by measuring the sorption isotherms at two different temperatures (namely 298 K and 273 K) for the three materials. At low pressure, despite the difficulty of interpolating the experimental adsorbed amounts, the calculated adsorption enthalpies were found to be very close, as reported in Table 2 (between −32 kJ·mol^−1^ to −34 kJ·mol^−1^ in the case of MIP-202). However, the enthalpic profiles are very different. In the case of MIP-202, the decrease in the heat of adsorption is very moderate compared to the other materials. This behavior could be attributed to the interaction between CO_2_ and amino functions or extra-framework anions, which would be a prominent sorption process, up to θ = ~0.4 where the isosteric curves become parallel. Looking at the sorption isotherm, the value of θ = ~0.4 corresponds to the inflexion mentioned above, which is the evidence that CO_2_ adsorbs according two different sorption processes. This difference confirms the role of the amino groups for the adsorption of CO_2_ as already demonstrated in the literature [55]. In the case of Muc-Zr MOF and MOF-801, if the enthalpy of adsorption is close to that of MIP-202 at low coverage, it decreases very drastically, which can be interpreted by the existence of very few sites of higher energy, which are saturated at very low coverage. These sites are likely the chemically unsaturated sites (CUS) borne by the Zr centres, a common feature to the materials explored in this work. Once these sites are saturated, adsorption takes place in the cavities of the materials, while in the case of MIL-202, sorption can take place on the extra-framework anions and amine function, thus justifying the slow decrease of the enthalpy of adsorption with coverage.

The theoretical enthalpies of adsorption obtained from Monte Carlo simulations are reported in Table 3. They are weaker, located between −19 and −23 kJ·mol^−1^ for Muc-Zr and MOF-801. The classical Monte Carlo calculations do not take into account the creation and deletion of chemical bonds and therefore fail to reproduce the chemisorption on the metal centers, which explains the differences observed between experimental and theoretical results. However, the experimental and modelled data are very close at saturation where physisorption is only observed. Interestingly, close to saturation, the enthalpy of adsorption remains higher in the case of MIP-202, which is likely the consequence of stronger confinement effects due to the smaller size of the cavities [49,56]. As Muc-Zr MOF has larger cavities, this confinement effect is lower as well as the enthalpy of adsorption of CO_2_. The lowest value is found for MOF-801, which is consistent with the existence of mesopores in this material. Additionally, it can be noted that, whatever the system, the enthalpy of adsorption lies not far above the enthalpy of liquefaction of CO_2_, which confirms a moderate sorbate/sorbent interaction. However, it remains above the enthalpy of liquefaction, even close to saturation, which is a consequence of the confinement effects.

From these results, it can be concluded that CO_2_ sorption is sensitive to two different affects (i) the presence of amino groups, which favors MIP-202 at the expense of the other MOFs and (ii) the size of the cavities in which CO_2_ is confined. Muconic and fumaric ligands do not have any specific interaction for CO_2_; however, the muconic ligand is longer, inducing larger cavities and, therefore, a weaker confinement effect. In other words, there is more room for carbon dioxide in these cavities, but a weaker interaction is involved. In addition, in MIP-202, the presence of extra-framework anions (Cl^−^) illustrated in the literature should also be considered as important adsorption sites if available [44]. Such conclusions can be supported by the snapshots obtained from Monte Carlo simulations and corresponding to the most probable positions of guest molecules in the pores (see Figure S4). It can clearly be observed on the snapshots that the CO_2_ molecules strongly interact with MIP-202 (mainly with NH_2_/NH_3_^+^ groups and Cl^−^), while they interact more weakly with MOF-801 and Muc-Zr (mainly with carboxylate groups and muconic/fumaric groups).

### 2.3. Ethanol Adsorption

**Figure 9:**
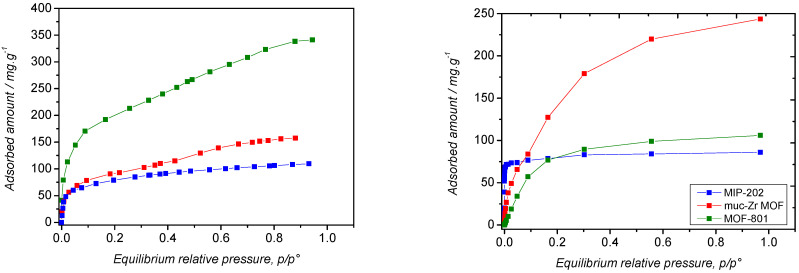

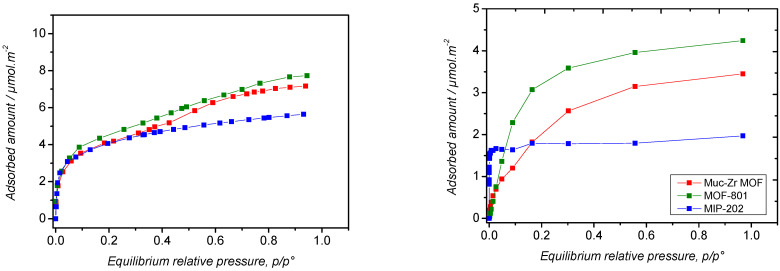
Ethanol sorption isotherms at 303 K by the prepared MOFs. (Red) Muc-Zr MOF, (green) MOF-801, (blue) MIP-202. (**Left**) Experimental data (**Right**) predicted data.

Regarding VOCs, which have higher boiling temperatures and therefore stronger interaction compared to CO_2_, we decided to focus on a rather simple model: ethanol. This molecule is able to interact through van der Waals interaction but also through hydrogen bonding. The sorption isotherms obtained at 303 K are reported in Figure 9 and the corresponding enthalpies of adsorption in Table 4.

The comparison between experimental and theoretical isotherms shows a relative good agreement in the amount adsorbed in the three solids, if we take into account the absence of mesopores for MOF-801 in simulations, as well as the presence of impurities in Muc-Zr-MOF. Theoretical saturations obtained from Monte Carlo calculations are in good agreement with the values of theoretical pore volumes and sizes obtained on the optimized structures.

As MIP-202 and MOF-801 have similar pore volumes and pore sizes, the saturation values for ethanol are also close. The comparison of the affinity of the solids for ethanol can be discussed using the adsorption enthalpy and the slope of the isotherms. It follows that the affinity increases as a function of the sequence: MIP-202 > Muc-Zr > MOF-801. As confirmed by the snapshots (Figure S4), the presence of Cl^−^ seems strongly enhance the interaction between ethanol and MIP-202, while the NH_2_ groups appear as a secondary interacting group in this case. Regarding Muc-Zr, the presence of carboxylate groups seems important in the interaction with ethanol.

**Figure 10:**
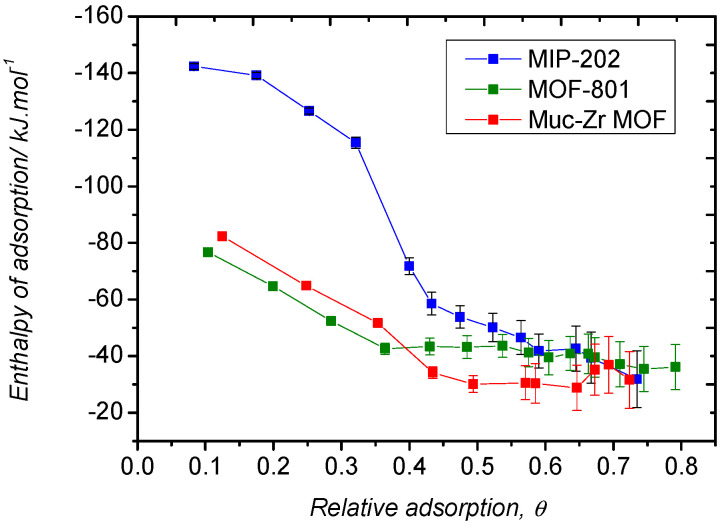
Enthalpic profiles obtained by microcalorimetry upon adsorption of ethanol by the three MOFs at 303 K.

The enthalpic profiles obtained by in situ microcalorimetry are shown in Figure 10. The general trends observed in the case of the adsorption of CO_2_ can be mentioned for ethanol. The enthalpy of adsorption obtained in the case of MIP-202 is higher at low coverage, which emphasizes the importance of the amino functions for the sorption of polarizable or acidic species [57].

It can be noted that, considering the error bars, the enthalpy are similar at saturation, that is, very close to the enthalpy of liquefaction of ethanol (−42.3 kJ·mol^−1^). However, the shapes of the enthalpic profiles are very different as they progressively decrease from very low coverage down to saturation for MOF-801 and Muc-Zr MOF, whereas, in the case of MIP-202, there is a sudden decrease from θ = ~0.35. This value is similar to that observed in the case of the adsorption of CO_2_, which suggests that ethanol and carbon dioxide species undergo the same sorption process on MIP-202. In this particular case, sorption would first occur on the extra-framework anions and amino functions borne by the aspartic ligands and only in a second stage could sorption occur in the cavities through van der Waals interaction. The observation that, in the case of MOF-801, the enthalpic curve at low coverage is found below that of Muc-Zr MOF is consistent with the fact that this material has a noticeable mesoporosity. Indeed, confinement effects decrease for larger pores, thus explaining the lowest value found for this material [50,51]. 

## 3. Conclusions

In this study, we have shown that polarisable molecules such as carbon dioxide and ethanol can be adsorbed by microporous zirconium-based MOFs. Two distinct sorption processes have been suggested for accounting for the enthalpic profiles exhibited by the different materials. In the case of MIP-202, in which amino groups are borne by the aspartic ligands, sorption first involves these amino groups (as well as extra-framework anions) from zero coverage. Once these sites are saturated, sorption can take place on less intense sites in the cavities of MIP-202. This scenario is well confirmed by modelling studies, despite the difficulty of taking chemisorption into account. In the case of MOF-801 and Muc-Zr MOF, which differ in terms of ligand length and therefore pore volume, similar results have been found, even though MOF-801 shows a higher sorption capacity, despite a shorter ligand. This has been rationalized by evidencing the presence of mesopores in MOF-801 that have unexpectedly appeared during its synthesis.

## 4. Materials and Methods

All the chemicals were purchased from Aldrich Sigma in France. Two zirconium sources were used: zirconium (IV) oxychloride octahydrate (ZrOCl_2_·8H_2_O, 99.5%) and zirconium tetrachloride (ZrCl_4_, 99.9%). Three different ligands were used: fumaric acid (C_4_H_4_O_4_, 99%), L-aspartic acid (98%) and trans, trans muconic acid (98%). We also used formic acid as a modulator (HCOOH, 95%), N, N dimethylformamide (DMF, 99%), ethanol (C_2_H_5_OH, 96%) and hydrochloric acid (35 wt%). As first mentioned by Kitagawa et al., the modulator approach consists in the addition of monovalent modulator molecules. It enhances the reproducibility of the synthesis procedures, allows increasing the crystallinity of the product and, in certain cases, to control the crystal size and morphology as well as the degree of agglomeration/aggregation of the crystals [58,59]. 

### 4.1. Preparation of MOF-801

MOF-801 was synthesized according to a published route [29] by mixing ZrOCl_2_ (8H_2_O) (3.2 g, 10 mmol) and fumaric acid C_4_H_4_O_4_ (1.16 g, 10 mmol) in the presence of dimethylformamide DMF (40 mL). Formic acid (14 mL, 1M) used as a modulator was introduced in the mixture, which was stirred for 30 min at room temperature. The mixture was put in an ultrasonic bath (80 °C, 45 kHz, 90 W). After 30 min of reaction, a white precipitate was recovered by centrifugation and washed three times daily for 3 days with DMF, and three times daily for 3 days with ethanol. The solid was activated by drying for 24 h at two different temperatures (150 °C and 180 °C) under secondary vacuum.

### 4.2. Preparation of MIP-202

Measures of 1.15 g of ZrCl_4_ and 1.40 g of L-aspartic acid were mixed in 10 mL of water and placed in a sealed flask under autogen pressure in a silicon oil bath at 110 °C for 24 h. The obtained product was recovered by centrifugation and washed first with water and then with ethanol three times and dried under secondary vacuum at room temperature for 24 h [40].

### 4.3. Preparation of Muc-Zr MOF

Measures of 1.6 g of ZrCl_4_ and 0.975 g of trans, trans muconic acid with 576 µL of HCl as modulator, were mixed in 48 mL of DMF, and put in a small sealed flask in a silicon oil bath at 150 °C for 24 h under autogen pressure. The obtained product was recovered by centrifugation and washed three times with acetone.

### 4.4. Characterization and Analytical Procedures

The crystalline phases of the MOF-801, MIP-202, and Muc-Zr MOF were characterized by X-ray powder diffraction using a RIGAKU MINIFLEX 600 diffractometer and a BRUKER France SAS AXS D8 ADVANCE diffractometer. Thermo-gravimetric analyses (TGA, TA Instruments SDT Q600 Thermal Gravimetric Analyzer, New Castle, DE, USA) were recorded using a ramp of 10 °C/min. The textural properties of the materials were characterized by nitrogen adsorption at 77 K using a micromeritics VacPrep 061 (sample degas system) and a micromeritics TriStar (Surface Area and Porosity Analyzer (TriStar 3000 gas adsorption analyzer (Micromeritics, Norcross, GA, USA). The equivalent specific surface areas were determined using the BET transform of the sorption isotherm in the relative pressure range between 0.005 and 0.05, taking 0.162 nm^2^ as the cross-sectional area for adsorbed nitrogen molecules. Scanning electron microscopy (SEM) was performed using a using a Hitachi S4800 microscope (Hitachi, Tokyo, Japan). The ultrasonic bath was an Elma transonic Ti-H-5 brand (Singen, Germany). Fourier transform infrared spectroscopy in transmission mode has been performed using a IFS55 Bruker spectrometer (Bruker, Paris, France) (MCT cryodetector) with a 2 cm^−1^ resolution in a spectral domain 6000–400 cm^−1^. The different powdered materials were diluted using dry KBr before being pressed and further analysed.

### 4.5. Sorption Studies

CO_2_ and ethanol adsorption measurements were performed at 298 K by using a device developed for this purpose. This apparatus is based on manometric measurements, using two capacitive pressure gauges (10 Torr and 1000 Torr). Detailed information on the device has been already described [60,61]. In a few words, the adsorbed amount is calculated from the pressure difference at the equilibrium before and after each adsorption step. In the case of carbon dioxide, the gas was readily obtained from a CO_2_ cylinder of high purity, whereas in the case of ethanol, the vapour was obtained from a dried liquid ethanol (treated with activated molecular sieve 5 Å) at the temperature of the sorption experiments (298 K). A quantity of gas is introduced in a calibrated volume where the equilibrium pressure is measured before adsorption. Once the pressure is stable, the gas is expanded in the sample cell where the pressure is recorded. Once the pressure is stable, the equilibrium pressure is used to derive the amount adsorbed. At these low pressures, the gases are supposed to behave as ideal gases. In commercial sorption machines, target equilibrium pressures are set in the sorption programme. In our apparatus, we decided to allow the sample to monitor itself the number of data useful to cover the whole sorption isotherm. In other words, after each sorption datum, additional gas is introduced in the calibration volume for the next measurement. If, after adsorption, the equilibrium pressure is very low, the initial pressure of the following point will be very close to that set for the previous point. In this way, any specific sorption process can be disclosed. Prior to measurements, about 200 mg of material was degassed at 120 °C for 12 h under a vacuum (10^−5^ Torr).

### 4.6. Molecular Simulations

Before probing the adsorption behavior of the three solids, density functional theory (DFT) calculations were used to optimize the crystal structures. Starting from the data available in the literature for MOF-801 and Muc-Zr (for modified UiO-66) [43,47], the DFT geometry optimization procedure was performed using DMol^3^ (from Accelrys), which uses a numerical radial function basis set to calculate the electronic properties of clusters and crystal solids from first principles. It has been followed to determine the structures and calculate the partial charges on each element [62]. For that purpose, using periodic models, the PW91 GGA density functional combined with the double numerical basis set containing polarization functions on hydrogen atoms (DNP) were considered [63]. In addition, all electrons have been taken into account. The convergence criteria for energy (10^−5^ Ha), maximal force (0.002 Ha Å^−1^), and maximal displacement (0.005 Å) allow the calculations to reach the global minimum. The partial charges have been calculated using the Mulliken scheme available in DMol^3^. They are reported in Figure S1.

Concerning MIP-202, the structure was optimized using the same procedure from before while the partial charges were directly taken from the literature [44]. The inclusion of extra-framework Cl^−^ was performed using the Monte Carlo simulations (with a partial charge fixed at −0.7).

From these partial charges obtained from DFT for each structure, electrostatic interactions were calculated by the Ewald summation method while short-range van der Waals interactions were evaluated using UFF force field containing Lennard Jones parameters submitted to a cut-off distance of 12 Å. Such forcefields for the solids combining electrostatic charges and Lennard Jones parameters were combined with forcefields for CO_2_ [64] and EtOH [65] to reproduce the intermolecular interactions and determine the adsorption properties for CO_2_ at 298 K and ethanol at 303 K. Grand Canonical Monte Carlo (GCMC) simulations were performed, making use of the SORPTION (from Materials Studio) or homemade code, based on the Metropolis Monte Carlo method, typically with 5.0 × 10^6^ Monte Carlo steps for production, following 5 × 10^6^ steps for equilibration [62]. From these calculations, it is possible to obtain (i) the adsorption enthalpy at low coverage illustrating the interactions between sorbate and sorbent, (ii) the adsorption isotherm up to the saturation or to the experimental maximum pressure, (iii) the distribution of the sorbate molecules upon the considered pressure and the global adsorption mechanism as a function of the textural and chemical properties of the investigated solids. Such a strategy has already proved to be efficient for the investigation of MOFs sorption behaviour [25,66,67].

## Figures and Tables

**Table 1:** Textural parameters after nitrogen adsorption at 77 K.

	Specific Surface Area (Langmuir)/m^2^·g^−1^	Pore Volume/cm^3^·g^−1^	External Surface Area/m^2^·g^−1^	Average Mesopores Diameter/Å
Muc-Zr MOF	462	0.157	14	-
MOF-801	920	0.230	205	68
MIP-202	405	0.09	106	-

**Table 2:** Theoretical values for specific surface area for N_2_ and pore volume for the investigated solids.

	Specific Surface Area/m^2^·g^−1^	Pore Volume/cm^3^·g^−1^
MIP-202	240	0.27
MOF-801	543	0.32
Muc-Zr MOF	1518	0.50

**Table 3:** Enthalpies of adsorption of CO_2_ by the three MOFs.

	Experimental Data		Simulated Data
	ΔH_ads_/−kJ·mol^−1^ Low Coverage	ΔH_ads_/−kJ·mol^−1^ *p* = 10^5^ Pa	ΔH_ads_/−kJ·mol^−1^ Low Coverage
Muc-Zr MOF	32	26	19
MOF-801	33	24	23
MIP-202	34	28	50
Enthalpy of condensation of CO_2_ at 288 K			16.7

**Table 4:** Enthalpies of adsorption of ethanol by the three MOFs.

	Experimental Data		Simulated Data
	ΔH_ads_/−kJ·mol^−1^ Extrapolation at Low Coverage	ΔH_ads_/−kJ·mol^−1^ *p* = 10^5^ Pa	ΔH_ads_/−kJ·mol^−1^ Low Coverage
Muc-Zr MOF	~100	~35	54
MOF-801	~90	~40	39
MIP-202	~145	~40	90
Enthalpy of condensation of ethanol at 298 K			42.3

## Data Availability

All the data are within the article and the Supplementary Materials.

## Supplementary Materials

The following are available online, Figure S1: Partial charges extracted from DFT geometry-optimizations, Figure S2: Transmittance infrared Spectra of the prepared materials, Figure S3: SEM image of MOFs, Figure S4: Snapshots obtained from Monte Carlo simulations and corresponding to the most probable adsorption sites for CO_2_ and EtOH.

## Sample Availability

The samples are no longer available.
