# Supplementary material for: Zirconium-Based Metal Organic Frameworks for the Capture of Carbon Dioxide and Ethanol Vapour. A Comparative Study
Source: Molecules. 2021 Dec 15;26(24):7620. doi: 10.3390/molecules26247620 (PMC8703343; doi:10.3390/molecules26247620)
Supplement: Supplementary file 1 [file molecules-26-07620-s001.zip › molecules-1504932-supplementary.pdf]

Zirconium based metal organic frameworks for the capture of carbon dioxide and ethanol vapour. A comparative study.

Meryem Saidi[1,2], Phuoc Hoang Ho[1], Pankaj Yadav[1], Fabrice Salles[1], Clarence Charnay[1], Luc Girard[3], Leila Boukli-Hacene[2], Philippe Trens[1*]

[1] ICGM, Univ. Montpellier, CNRS, ENSCM, Montpellier, France

[2] Department of chemistry, Tlemcen University, Algeria

3. ICSM, CEA, CNRS, Marcoule, France

Supporting information.

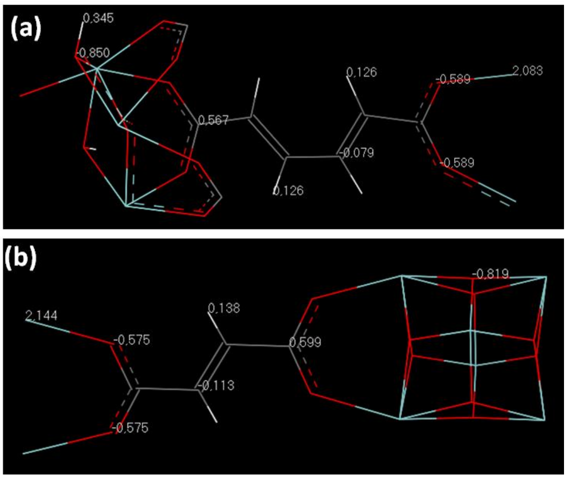

Figure S1. Partial charges extracted from DFT geometry-optimizations for Muc-Zr (a) and MOF-801 (b)

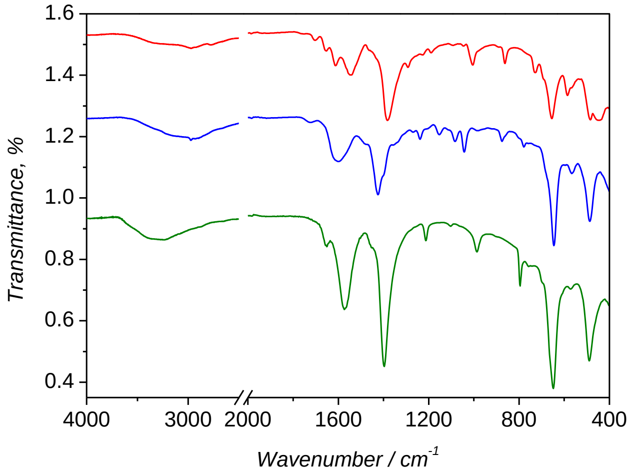

Figure S2. Transmittance infrared Spectra of the prepared materials. **(red)** MOF Zr-muconic, **(blue),** MOF MIP-202 and **(green)** MOF-801.

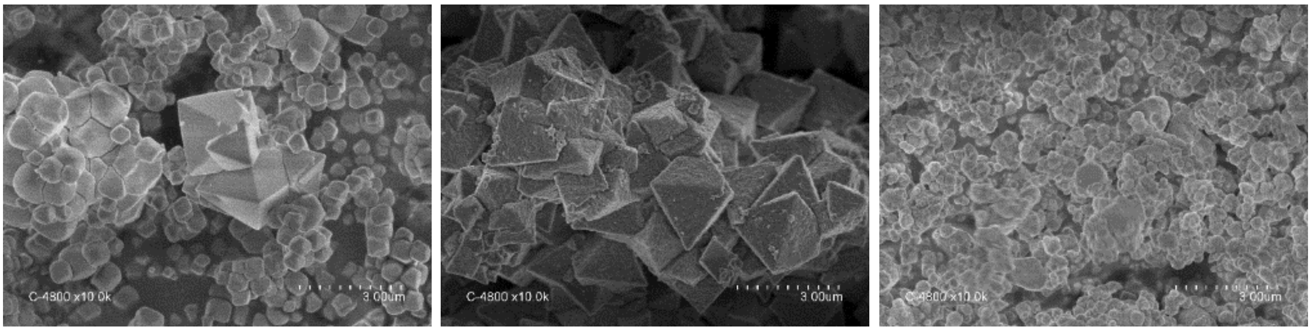

Figure S3. SEM image of MOFs: **(left)** MOF801 ,**(middle)** muc-Zr MOF, **(right)** MIP202.

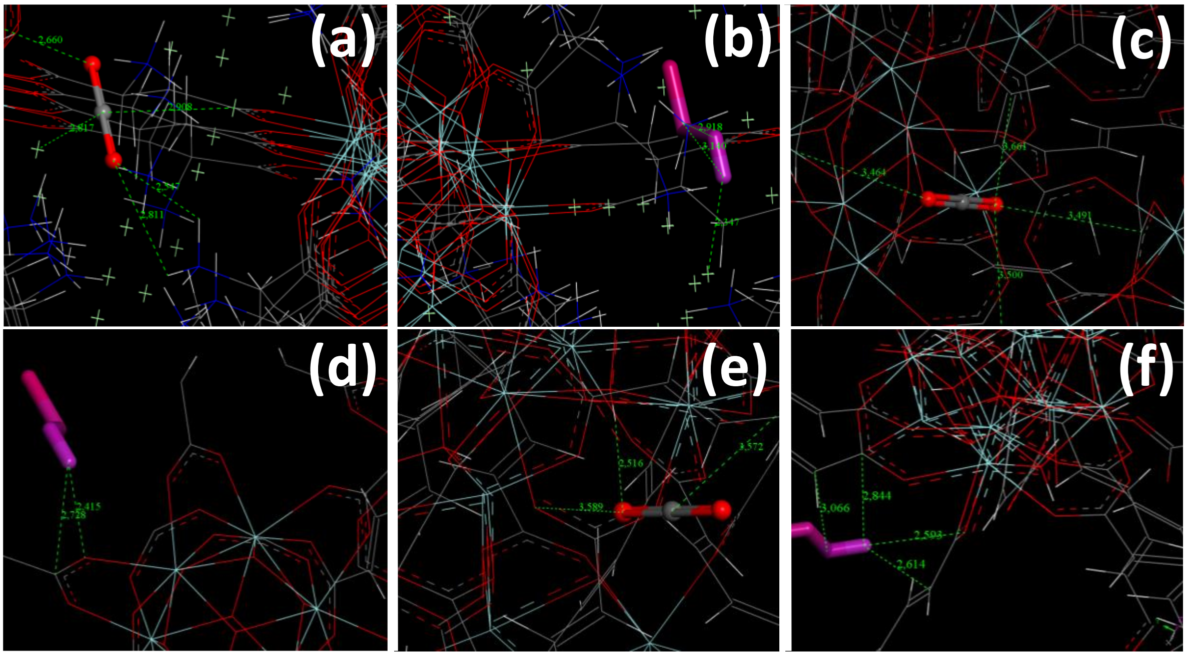

Figure S4. Snapshots obtained from Monte Carlo simulations and corresponding to the most probable adsorption sites for CO_2 and EtOH respectively in MIP-202 (a and b), MOF-80 (c and d) and Muc-Zr (e and f). The colors of the different atoms are C (grey), O (red), H (white), Cl (green), Zr (cyan), N (blue).
